# Glutamate’s Effects on the N-Methyl-D-Aspartate (NMDA) Receptor Ion Channel in Alzheimer’s Disease Brain: Challenges for PET Radiotracer Development for Imaging the NMDA Ion Channel

**DOI:** 10.3390/molecules29010020

**Published:** 2023-12-19

**Authors:** Nehal M. Shah, Nane Ghazaryan, Noresa L. Gonzaga, Cayz G. Paclibar, Agnes P. Biju, Christopher Liang, Jogeshwar Mukherjee

**Affiliations:** Preclinical Imaging, Department of Radiological Sciences, University of California-Irvine, Irvine, CA 92697, USA; nmshah303@gmail.com (N.M.S.); ghazaryn@uci.edu (N.G.); nlgonzag@uci.edu (N.L.G.); cgpaclib@uci.edu (C.G.P.); apbiju@uci.edu (A.P.B.); liangc@uci.edu (C.L.)

**Keywords:** NMDA ion channel, glutamate, MK-801, memantine, Alzheimer’s disease, PET imaging, Aβ plaques, Tau

## Abstract

In an effort to further understand the challenges facing in vivo imaging probe development for the N-methyl-D-aspartate (NMDA) receptor ion channel, we have evaluated the effect of glutamate on the Alzheimer’s disease (AD) brain. Human post-mortem AD brain slices of the frontal cortex and anterior cingulate were incubated with [^3^H]MK-801 and adjacent sections were tested for Aβ and Tau. The binding of [^3^H]MK-801 was measured in the absence and presence of glutamate and glycine. Increased [^3^H]MK-801 binding in AD brains was observed at baseline and in the presence of glutamate, indicating a significant increase (>100%) in glutamate-induced NMDA ion channel activity in AD brains compared to cognitively normal brains. The glycine effect was lower, suggesting a decrease of the co-agonist effect of glutamate and glycine in the AD brain. Our preliminary findings suggest that the targeting of the NMDA ion channel as well as the glutamate site may be appropriate in the diagnosis and treatment of AD. However, the low baseline levels of [^3^H]MK-801 binding in the frontal cortex and anterior cingulate in the absence of glutamate and glycine indicate significant hurdles for in vivo imaging probe development and validation.

## 1. Introduction

The glutamate excitatory system in the brain functions using metabotrophic glutamate receptors (mGlu) and ionotrophic receptors (iGlu). The ionotrophic receptors have been of much interest due to their prominent role in long-term potentiation (LTP) and neurodegeneration [1]. The ionotrophic receptors are classified based on their agonist selectivity into AMPA (α-amino-3hydroxy-5-methyl-4-isoxazolepropionic acid), Kainate and NMDA receptors. The NMDA receptor is a major receptor subtype for glutamate and aspartate in the mammalian central nervous system [2]. The ion channel of the NMDA receptor is permeable to calcium ions and is responsible for synaptic plasticity [3]. It is also the cause of neuronal death under excitotoxic pathological conditions [4]. The pharmacological role of NMDA receptors has drawn an increased amount of attention as it is believed to be involved in various brain physiological and pathological processes such as ischemia [5], epilepsy [6], stroke, traumatic brain injury, hypoglycemia [7], depression [8], schizophrenia [9], Parkinsons disease [10], Alzheimer’s disease (AD) [11], and Huntington’s chorea [12]. In addition, the NMDA receptor has been shown to be essential for neuronal and behavioral plasticity, and hence has effects on learning and memory processes in the development of brain [13].

**Figure 1:**
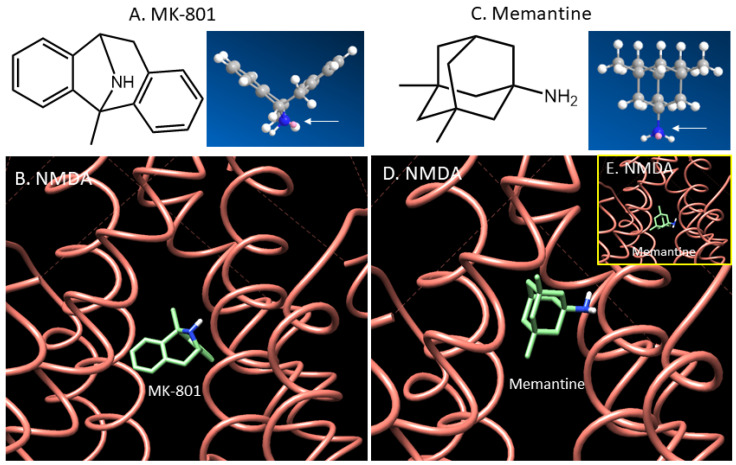
Comparison of MK-801 and memantine binding to the NMDA ion channel: (**A**,**B**). MK-801 with .mol structure (arrow showing nitrogen atom in dark blue) docked to human GluN1/GluN2A NMDA receptor (PDB 6IRH) using Chimera AutoDock. Binding energy for MK-801 was −7.3 Kcal/mol. (**C**–**E**). Memantine with .mol structure (arrow showing nitrogen atom in dark blue) docked to human GluN1/GluN2A NMDA receptor (PDB 6IRH) using Chimera AutoDock. Preferred binding site of memantine was somewhat higher than MK-801 with binding energy of −6.7 Kcal/mol (inset shows binding of memantine at the same MK-801 site with binding energy of −5.6 Kcal/mol).

The NMDA ion channel can be blocked by high-affinity drugs such as MK-801 (Ki = 2.5 nM [14]) but these would cause unacceptable side-effects (Figure 1A,B). Therefore a moderate/lower-affinity drug such as memantine (Ki = 0.69 μM [15]) has been used in moderate to severe AD (Figure 1C–E). Using Chimera Autodock, we found a weaker binding of memantine compared to MK-801 in the ion channel with minor differences in the binding site (Figure 1). This binding profile of both MK-801 and memantine is similar to previously reported docking of the drugs to the NMDA ion channel [16,17].

The NMDA receptor is highly concentrated in the hippocampus and cortical regions. Modest amounts are found in the striatum and other brain regions. The cerebellum has the lowest amount of the receptors and may therefore be able to serve as a reference region for imaging studies [18]. Hippocampal NMDA receptors have been studied extensively for their long-term potentiation and role in learning and memory [13]. Glutamate and glycine together as co-agonists facilitate the opening of the NMDA receptor ion channel for calcium influx into the neurons [19]. Memantine has been shown to be efficacious for use in AD, perhaps because of its uncompetitive fast off-rate mechanism acting on the extrasynaptic NMDA receptor ion channels [20]. The recent elucidation of the crystal structure of the NMDA receptor [15] has provided more detail on the interactions of the ligands with various sites [14,21,22].

Alzheimer’s disease (AD) brains exhibit extensive cellular atrophy and cell loss, a shrinkage of cortical thickness, an enlargement of sulci and ventricles and changes in multiple neurochemical systems. In addition to the presence of senile plaques (SP) and neurofibrillary tangles (NFT) in the AD brain, there is a need to identify complimentary biochemical processes that may aid in the early diagnosis and development of therapeutics for AD. A number of studies have implicated the NMDA ion channel in AD [23], and memantine has been FDA-approved for use in AD [24]. Thus, successful in vivo imaging methods used to study NMDA ion channel activity will have great value.

**Figure 2:**
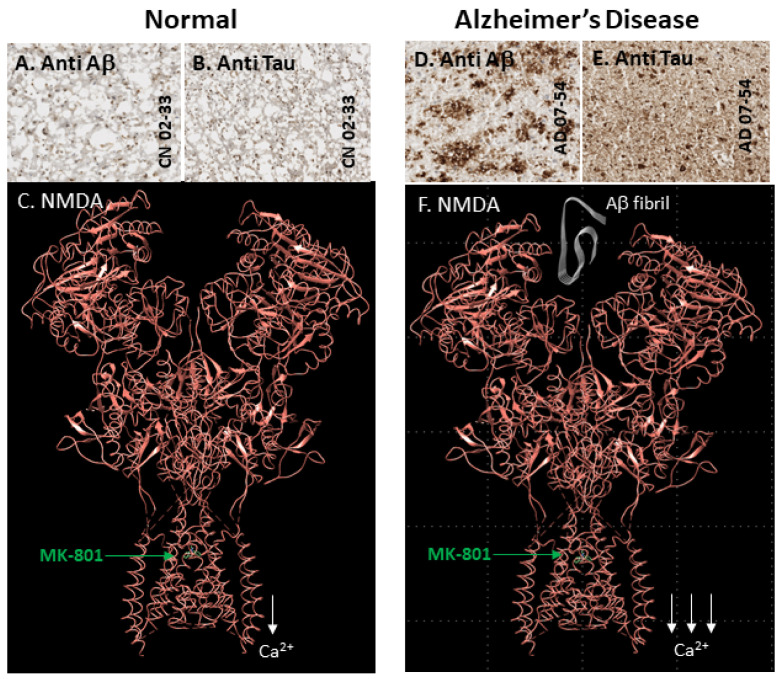
Structure of NMDA receptor: (**A**–**C**) human GluN1/GluN2A NMDA receptor (PDB 6IRH) complex molecular model illustrating normal ion channel activity and binding of MK-801 in the ion channel (green); (**D**–**F**) human GluN1/GluN2A NMDA receptor (PDB 6IRH) complex model in the presence of AD pathology (Aβ plaques and Tau). For simplicity, Aβ fibril is shown interacting with the ion channel to cause it to remain open for extended periods, enabling a greater influx of calcium ions. The ion channel thus has more available binding sites for MK-801 and memantine.

Because measuring NMDA ion channel activity involves the binding of the imaging probe to “open channels” [25], approaches to the development and evaluation of diagnostic probes for this biomarker for in vivo use are different, compared to traditional receptors [26] or other ion channels where the binding site is on the surface of the protein [27]. Aside from issues of binding affinity, lipophilicity, the free fraction in plasma, blood–brain barrier (BBB) permeability and metabolic stability, the “availability” of the binding site is crucial. The evaluation of these “available” sites can become difficult if the number of open channel binding sites is low in the normal state. Figure 2A–C show a normal state of the NMDA receptor, where the open channel (to which MK-801 can bind) allows calcium ion to flow through. The availability of open ion channels may be increased by the agonist glutamate (which does not cross the BBB) or in pathology (Figure 2D–F show an AD brain where the presence of Aβ plaques and Tau may alter the functional status of the ion channel). 

Glutamate was found to open ion channels in brain homogenates and thus exhibit a higher glutamate-induced [^3^H]MK-801 binding with lower effects of glycine [28]. Abnormal NMDA ion channel activity has been reported previously in AD [29], but in vivo imaging of this altered function has not been possible. Many efforts have been made over the last few decades, without success. More recently, successful PET radiotracers have been developed for an alternate GluN2B binding site on the NMDA complex [30]. The sensitivity of this binding site to NMDA ion channel activity remains to be determined. Our efforts have been focused on identifying potential neurotransmitter receptor systems and gated ion channels that may be affected in AD and therefore serve as biomarkers for possible early diagnosis and treatment strategies. 

Thus, in this article, we have examined glycine- and glutamate-induced [^3^H]MK-801 binding in post-mortem AD and cognitively normal (CN) subjects’ brains (frontal cortex and anterior cingulate cortex) to understand extent of “available” ion channel sites under baseline conditions (no added neurotransmitters) and in the presence of glycine and glutamate. In light of the challenges posed for probe development, we make an assessment of the process of new probe evaluation for this ion channel and steps that may be necessary for its translation into human studies. 

## 2. Results

**Figure 3:**
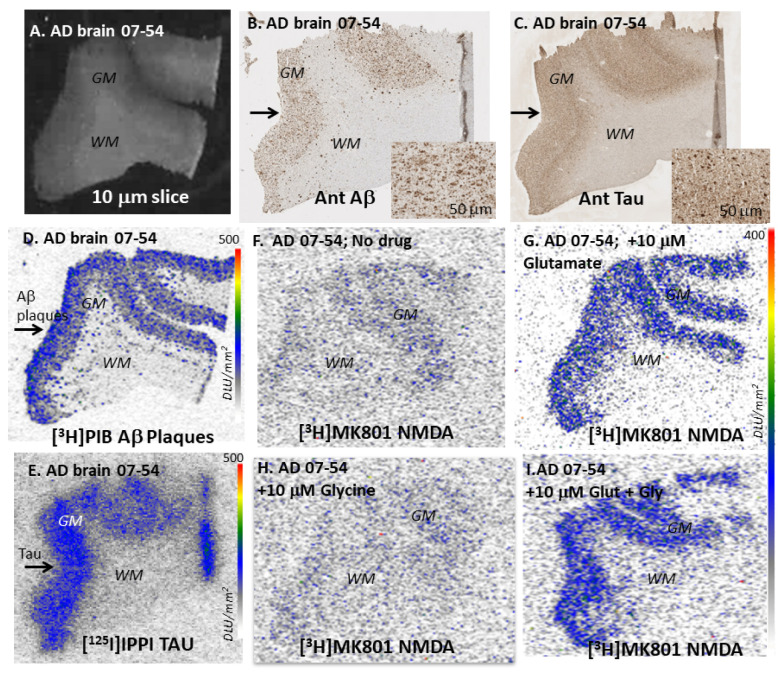
Post-mortem human AD brain, frontal cortex section: (**A**). AD brain frontal cortex slice showing gray (GM) and white matter (WM); (**B**). Anti-Aβ immunostain of adjacent section showing (arrow in GM) Aβ plaques; (**C**). Anti-Tau immunostain of adjacent section showing (arrow in GM) abundant Tau; (**D**). [^3^H]PIB binding to Aβ plaques (arrow) in GM regions [31]; (**E**). [^125^I]IPPI binding to Tau (arrow) in GM regions [31]; (**F**). Baseline (no drug) [^3^H]MK-801 adjacent to brain slice; (**G**). [^3^H]MK801 binding, in the presence of 10 μM glutamate, to adjacent slice, showing increased binding to GM regions; (**H**). [^3^H]MK801 binding, in the presence of 10 μM glycine, to adjacent slice, showing binding to GM regions; (**I**). [^3^H]MK801 binding, in the presence of 10 μM glutamate and 10 μM glycine, to adjacent slice, showing increased binding to GM regions.

The autoradiography results of one AD subject’s frontal cortex are shown in Figure 3A–I. Grey matter and white matter are easily distinguishable in Figure 3A. Adjacent brain slices were immunostained with anti-Aβ (Figure 3B) and anti-Tau (Figure 3C) to confirm presence of Aβ plaques and neurofibrillary tangles (NFT). Additionally, the extensive labeling of the grey matter with [^3^H]PIB in Figure 3D further confirmed the presence of Aβ plaques in those regions with the immunostaining of this AD subject (Figure 3B). Similar presence of [^3^H]PIB in all AD subjects’ frontal cortex and lack of [^3^H]PIB in control subjects was ascertained and was consistent with the information received with the brain tissues from Banner Health. For labeling Tau, we used [^125^I]IPPI (Figure 3E), which selectively labeled and confirmed the presence of Tau in adjacent brain slices of all AD subjects, consistent with the immunostain in Figure 3C [31].

The binding of [^3^H]MK-801 was seen in the gray matter regions of the frontal cortex brain slices of all the subjects. Compared to the [^3^H]MK-801 binding seen in the AD brain slice for the baseline (Figure 3F), the binding with added glutamate (Figure 3G) was significantly increased. Little activity was seen in non-specific binding slices, glutamate+MK-801, suggesting the specific binding of [^3^H]MK-801 to the NMDA ion channel. At baseline, the AD frontal cortex’s binding was 1.7 times higher than that of control brains. Added glutamate showed a 1.8-fold increase in binding in control subjects, while AD frontal cortexes showed a 2.8-fold increase compared to baseline. Thus, in the presence of glutamate, the AD frontal cortex’s binding increased significantly (>2.6-fold) compared to controls. In the presence of glycine, control subjects showed a greater increase in binding (>2-fold), while AD brains were not affected. However, this difference with glycine was suppressed in the presence of glutamate. These results indicate a significant increase in glutamate-induced [^3^H]MK-801 binding in AD brains, while little change was seen in the presence of glycine. This reduced glycine effect in AD brains has been previously reported [28].

**Figure 4:**
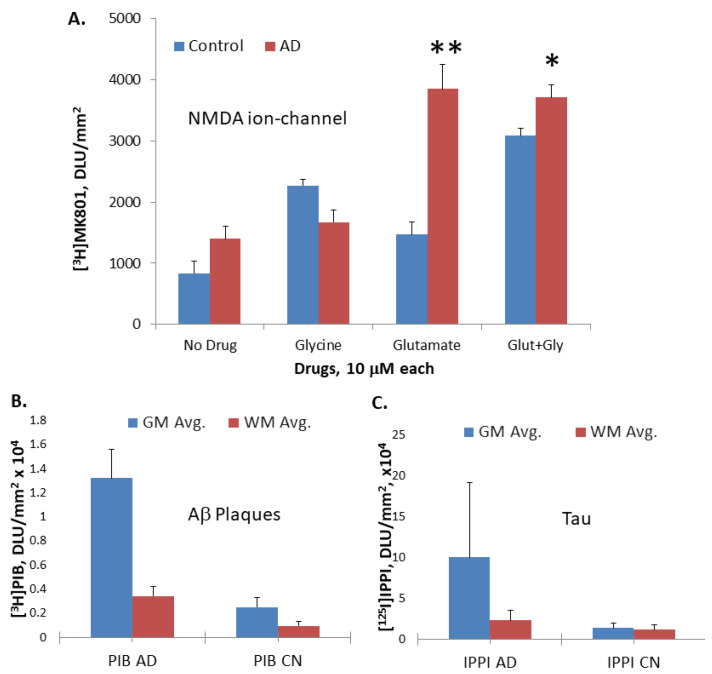
Post-mortem human AD and CN brain section analysis: (**A**). Plot of [^3^H]MK801 binding of all AD and control subjects’ GM frontal cortex regions under different drug conditions, Glu: glutamate; Gly: glycine (* *p* < 0.05; ** *p* < 0.01); (**B**). Plot of [^3^H]PIB in AD and control subject frontal cortex, showing high levels of Aβ plaques in AD [31]; (**C**). Plot of [^125^I]IPPI in AD and control subject frontal cortex, showing high levels of Tau in AD [31].

Our findings show a significant increase in glutamate-induced [^3^H]MK801 binding in AD brains, while little change was seen in the presence of glycine (Figure 4A). While it is speculated that the excess influx of calcium from this hyperactivation of the NMDA ion channel causes excitotoxic effects and leads to neuronal death in AD [32], it has yet to be proven or confirmed. Nonetheless, such a dramatic increase in binding with added glutamate in AD brains, with reduced effects with added glycine, the co-agonist, suggests a potential anomaly in the glycine site, as well as in the glutamate site in AD. Both of these binding sites may be affected by the presence of Aβ plaques and Tau (Figure 4B,C) in these AD subjects.

**Figure 5:**
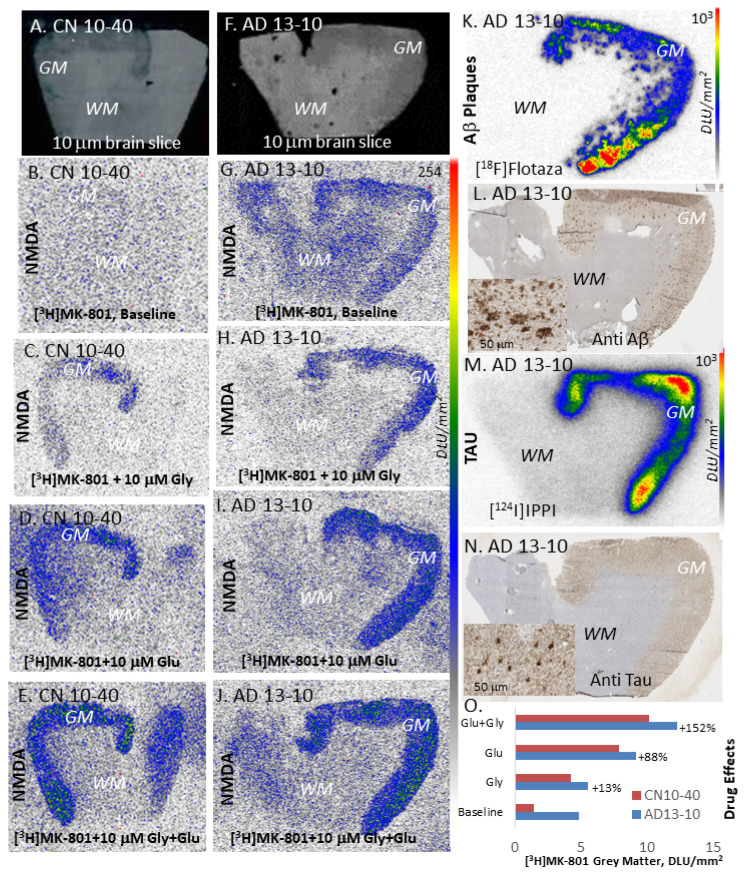
Post-mortem human AD brain, anterior cingulate section: (**A**). CN brain, anterior cingulate brain slice showing gray (GM) and white matter (WM); (**B**–**E**). [^3^H]MK801 binding in baseline (no drug), 10 μM glycine, 10 μM glutamate and in the presence of 10 μM glycine + 10 μM glutamate to adjacent slice, showing increased binding to GM regions; (**F**). AD brain, anterior cingulate brain slice showing gray (GM) and white matter (WM); (**G**–**J**). [^3^H]MK801 binding in baseline (no drug), 10 μM glycine, 10 μM glutamate and in the presence of 10 μM glycine + 10 μM glutamate to adjacent slice, showing increased binding to GM regions; (**K**). [^18^F]Flotaza binding to Aβ plaques in GM regions [33]; (**L**). Anti-Aβ immunostain of adjacent section showing Aβ plaques; (**M**). [^124^I]IPPI binding to Tau in GM regions [34]; (**N**). Anti-Tau immunostain of adjacent section showing abundant Tau; (**O**). Plot showing increased GM binding of [^3^H]MK-801, Glu + Gly > Glu > Gly > baseline, to available NMDA ion channel sites under different drug conditions in CN and AD brains.

Similar findings of the binding of [^3^H]MK-801 were observed in the anterior cingulate brain of AD slices (Figure 5). At baseline, in AD subjects (Figure 5G), the binding of [^3^H]MK-801 to the grey matter regions was low, with a ratio of GM/WM ~ 1.2. However, with the addition of glutamate and glycine (Figure 5J), a significant increase (152%) in the binding was observed compared to the baseline. Glycine alone had a small effect (Figure 5H), while glutamate alone had a larger effect (88%; Figure 5I,O). Adjacent slices of the same AD subject confirmed the presence of abundant Aβ plaques (Figure 5K,L) and Tau (Figure 5M,N). More significantly, as noted in the frontal cortex, at baseline, AD brains (Figure 5G) had more open NMDA channel availability compared to CN brains (Figure 5B), as reflected by their greater [^3^H]MK-801 binding (Figure 5O). 

## 3. Discussion

Even with the presence of abundant Aβ plaques and NFT in the frontal cortex and anterior cingulate of the AD subjects reported in this study, the NMDA ion channel was found to be very responsive to the neurotransmitters glycine and glutamate and would be considered “functional”, similar to the CN brain samples from these two brain regions. In both CN and AD subject brains, the binding of [^3^H]MK-801 was found to be low in the absence of any neurotransmitters, suggesting the “low availability” of the ion channel (Figure 3 and Figure 5). This low availability of the ion channel at baseline conditions poses a significant challenge in the development of positron emission tomography (PET) and single-photon emission computed tomography (SPECT) imaging probes for the NMDA ion-channel.

Our results indicate a significant increase in glutamate-induced [^3^H]MK801 binding in AD brains, while a smaller effect was seen in the presence of glycine, suggesting an anomalous glycine site in AD. These findings are consistent with several reports published previously on [^3^H]MK-801 [28], including the finding of some variability in different brain regions. It has been previously reported that fragments of the Aβ peptide may have moderate affinity for the agonist binding sites of the NMDA receptor [35]. This may be a possible reason for the anomalies seen at both the glutamate and glycine sites of the NMDA receptor. A possible ligand “locking” at the co-agonist glycine site by Aβ plaques in AD may possibly result in the hyperactivation by glutamate alone seen in our experiments. Other effects from neurofibrillary tangles as well as conformational effects on the NMDA ion channel in the AD brain may also lead to functional differences.

Based on the previously reported studies and our findings, at least two potential targets both for diagnostic purposes and therapeutic drug development in AD would be 1. the NMDA ion channel and 2. the glutamate site. Memantine is approved for moderate to severe cases of AD, as it binds to extrasynaptic NMDA receptor ion channels with low potency, with affinity in the micro-molar range rather than the nanomolar [16,36]. Therapeutic drugs, possibly antagonists, which may be able to modulate the glutamate site and decrease ion channel activity, will be helpful. From an imaging point of view, memantine or its radiolabeled analogs and radiolabeled analogs of MK-801 have not been successful for the in vivo imaging of the NMDA ion channel. 

**Figure 6:**
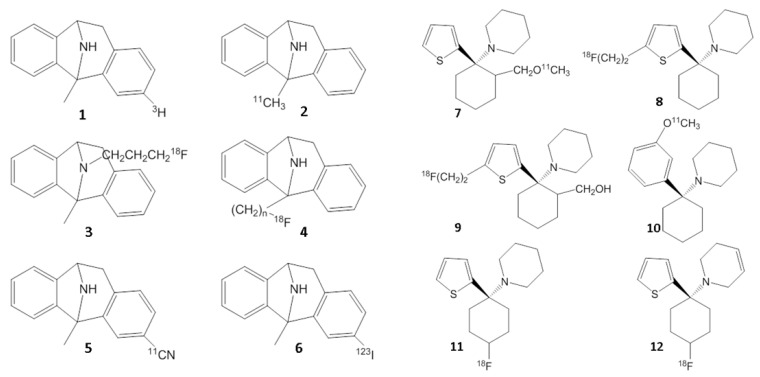
Chemical structures of [^3^H]MK-801 (**1**), PET radiotracers based on MK801 (**2**–**5**) and SPECT radiotracer based on MK-801 (**6**). Chemical structures of PET radiotracers based on PCP and TCP (**7**–**12**).

**Figure 7:**
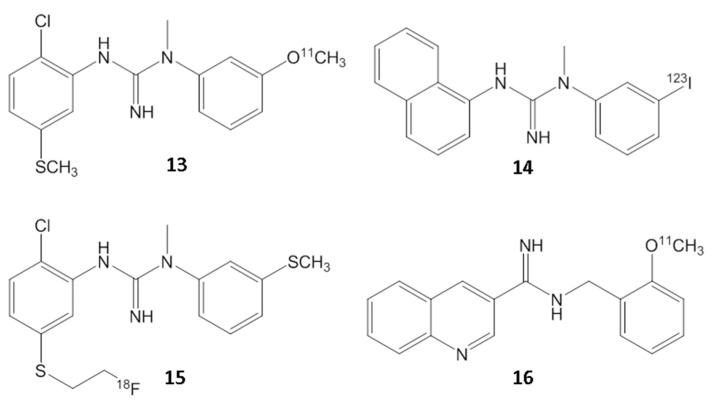
Radiolabeled carbon-11 (**13**,**16**), fluorine-18 (**15**) and iodine-123 (**14**) guanidine analogs investigated for the NMDA ion channel.

Despite the long-standing significance of the NMDA receptor complex, in vivo imaging of the ion channel has not yet been successful. The three primary target sites on the NMDA receptor complex include the glutamate binding region, glycine binding region and the ion channel binding region. None of these sites have been successfully imaged in vivo. There has been great interest in the development of radiotracers for the in vivo study of NMDA receptors using PET. The demonstration of the specificity of in vivo binding to the NMDA ion channel of these radiotracers has been a major challenge due to factors such as high non-specific binding, moderate affinity, fast in vivo kinetics and others. Efforts have been made towards [^18^F]- and [^11^C]labeled analogs of (PCP, TCP, MK-801), however, the in vivo localization of these radiotracers to the NMDA ion channel has proven to be difficult [37,38]. In order to minimize nonspecific binding (observed with [^123^I]IodoMK801 SPECT [39] and [^11^C]CN-MK801 PET [40]) PET imaging over an extended period is essential. In vivo imaging of the NMDA ion channel has not been able to successfully delineate the known distribution of the receptors using this class of radiotracers. Other radiotracer efforts include ^11^C-CNS 5161 [41], MK-801 derivatives [39,42,43], TCP derivatives [38], ^18^F-memantine [44] and ^11^C-AcL703 (glycine site; [45]). In vivo imaging of the NMDA ion channel has not been successful with these classes of radiotracers (Figure 6 and Figure 7).

Substituted guanidine derivatives such as [^18^F]GE-179 have been investigated as potential PET imaging agents for the NMDA ion channel [46,47]. However, the demonstration of the in vivo selectivity of binding with this class of compounds also has been difficult and has limited their use [48]. Although a number agonist and antagonist ligands are available for the glutamate and the glycine site of the NMDA receptor complex, no suitable in vivo radiotracer exists, primarily due to the highly ionic nature of the compounds, limiting their brain permeability.

**Figure 8:**
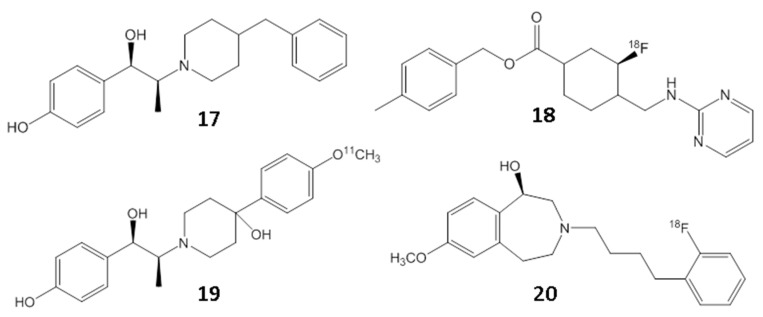
Chemical structures of GluN2B, showing ifenprodil (**17**) and the fluorine-18 (**18**) and carbon-11 (**19**) PET radiotracers derived from it. The related benzazepine derivative [^18^F]**20** has been successfully used for PET imaging studies for the GluN2B subunit.

Besides targeting the NMDA ion channel, efforts are also underway to target the GluN2B subunit of the NMDA receptor complex. Significant progress has been made on PET radiotracers for GluN2B. However, even this target has proved to be a difficult challenge until recently [49,50]. There has now been success in the in vivo imaging of the GluN2B subunit by using a benzazepine derivative, ((*R*)-[^18^F]-OF-Me-NB1; [^18^F]**20**, Figure 8; [51]). Although this is promising, the clinical significance of the GluN2B site is under study, since none of the drugs for this site have shown efficacy in animal models. The ability to image GluN2B will provide supporting evidence for the presence or absence of GluN2B containing NMDA ion channels. More studies are required to understand the role of this binding site in the functionality of the channels. Since the NMDA ion-channel opening changes based on the presence or absence of neurotransmitters, such as glutamate and glycine, it will be important to understand if the binding of PET radiotracers at the GluN2B are able to detect the change in the NMDA ion-channel opening.

**Figure 9:**
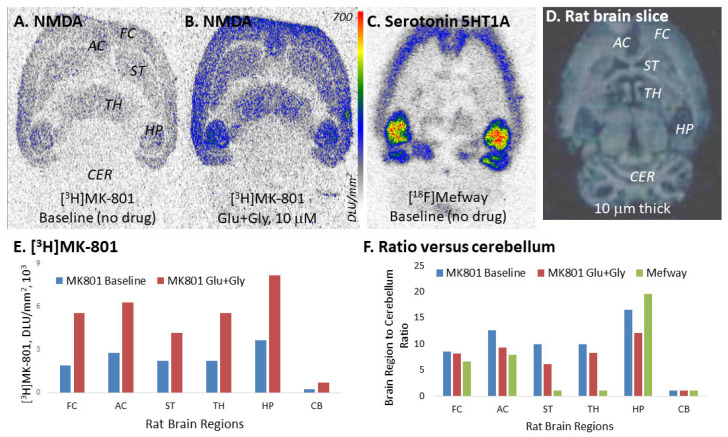
Rat brain [^3^H]MK-801 and [^18^F]Mefway comparison: (**A**). Normal rat brain, horizontal brain slice showing baseline (no drug) binding of [^3^H]MK-801; (**B**). Normal rat brain, horizontal brain slice showing binding of [^3^H]MK-801 in the presence of glutamate (Glu, 10 μM) and glycine (Gly, 10 μM); (**C**). Normal rat brain, horizontal brain slice showing baseline (no drug) binding of [^18^F]Mefway to serotonin 5-HT1A receptors; (**D**). Scan of normal rat brain, horizontal brain slice 10 μm thick; (**E**). Plot showing increased binding of [^3^H]MK-801 to available NMDA ion channel sites in the presence of Glu + Gly, compared to baseline; (**F**). Plot showing ratio of brain regions versus cerebellum for [^3^H]MK-801 binding to NMDA ion channels and [^18^F]Mefway to serotonin 5-HT1A receptors (FC: frontal cortex; AC: anterior cingulate; ST: striatum; TH: thalamus; HP: hippocampus; CB: cerebellum).

Figure 9 shows [^3^H]MK-801 binding in rat brain slices under baseline conditions (Figure 9A) and in the presence of glutamate and glycine (Figure 9B). The extent of the binding was compared with [^18^F]Mefway, a serotonin 5-HT1A receptor (Figure 9C), which has been successfully used in human PET studies [26]. Glutamate and glycine increased the binding of [^3^H]MK-801 by over 100% in all brain regions, including the cerebellum, consistent with previous reports (Figure 9E) [52]. However, when comparing ratios using the cerebellum (which is typically done in PET studies), they were found to be similar in the baseline and drug-induced conditions of [^3^H]MK-801 (Figure 9F). Importantly, [^18^F]Mefway, which binds to the G-protein coupled receptor, had similar ratios but has been successfully translated to human PET studies (Figure 9F) [26].

Since a direct comparison between in vitro findings and in vivo expectations cannot be made, at least two factors may be at play when comparing baseline of [^3^H]MK-801 (Figure 9A) with baseline of [^18^F]Mefway (Figure 9C): 1. Low target binding of MK-801 derivatives at baseline, and 2. High non-specific binding in vivo of MK-801 derivatives. These two factors together make measurement of “target engagement” difficult. It should be noted that glutamate and glycine levels in vivo vary across different brain regions, which may affect the extent of the binding of MK-801-based radiotracers, making it challenging to delineate changes with high sensitivity even with long half-life radiotracers such as [^123^I]IodoMK-801 [39].

**Figure 10:**
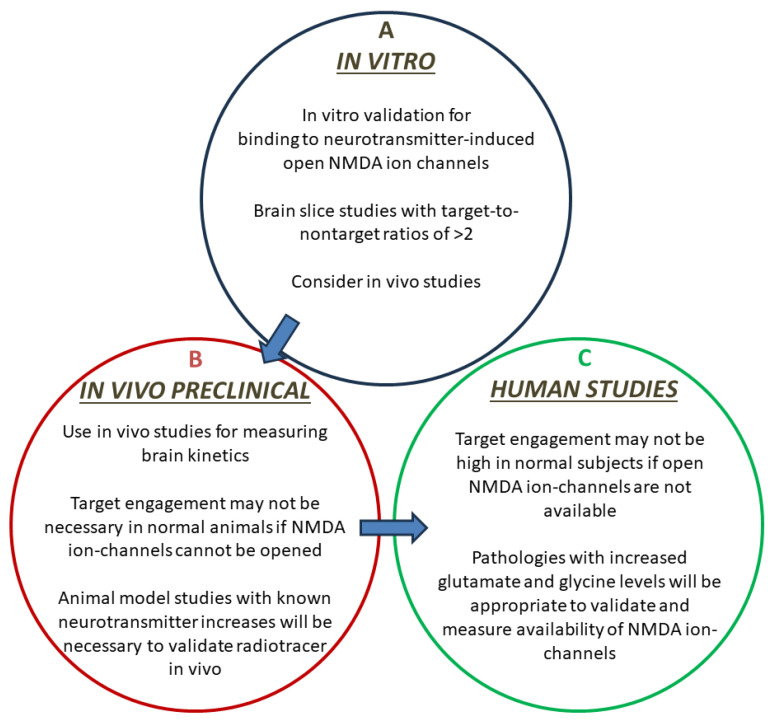
Potential strategy for in vivo radiotracer development for the NMDA receptor ion channel. (**A**). In vitro phase of radiotracer development including demonstration of binding sensitivity to glutamate and glycine-induced increased availability of ion-channels; (**B**). In vivo preclinical phase of testing the radiotracer in animal model studies with known brain fluctuations in glutamate and glycine concentrations; (**C**). Translation of selected radiotracer to human feasibility studies to assess increased “availability” of NMDA receptor ion-channels.

Our results and previous reported findings indicate that NMDA ion-channel activity is increased significantly in control and AD brains in the presence of glutamate [29,53]. A strategy to assess potential radiotracers for the NMDA ion channel, as outlined in Figure 10, has to take this “neurotransmitter-induced” radiotracer binding factor into account. Small animal models that may possess altered ion channel activity (due to elevated glutamate and glycine levels) will be important to assess the feasibility of radiotracers to measure in vivo open NMDA receptor ion channels [54,55,56]. It is very likely that this factor of non-availability of open ion-channels resulted in discarding potential radiotracers, which otherwise may have been useful [38].

Although an NMDA ion channel blocker, memantine, is used therapeutically in advanced AD [20,36,57], our findings suggest that the diagnostic and therapeutic targeting of the ion channel, glutamate site and glycine site may be appropriate in further refining and complementing the diagnosis and treatment of mild to severe AD [58,59]. Radiolabeling approaches with carbon-11, fluorine-18 and iodine-123/124 could be pursued to demonstrate their feasibility.

## 4. Materials and Methods

### 4.1. General Methods

[^3^H]MK801 and [^3^H]PIB were purchased from American Radiolabeled Chemicals, St. Louis, MO, USA. Unlabeled MK-801, glutamate, glycine and other biochemicals were purchased from Sigma-Aldrich, St. Louis, MO, USA.

### 4.2. Post-mortem Human Brain

Well-characterized frozen brain samples were obtained from BHRI, Sun City, Arizona [60]. The brain slices contained either the frontal cortex or the anterior cingulate, along with corpus callosum regions (total CN, *n* = 12; ages 81–90, and total AD, *n* = 12; ages 64–89). Brain sections were stored at −80 °C. Brain slices (10 µm thick) were obtained using a Leica 1850 cryotome. All post-mortem human brain studies were approved by the Institutional Biosafety Committee of the University of California-Irvine and were consistent with federal guidelines.

### 4.3. Rat Studies

Male Sprague Dawley rats, aged 8–10 weeks, were used in this study. Rats were purchased from Jackson Laboratories (Bar Harbor, ME, USA) and housed under controlled temperatures of 22 °C ± 1 °C, in a 12 h light–dark cycle, on at 6:00 AM, with water and food chow ad libitum. Isoflurane was used for euthanasia and the brains excised. Brain slices (10 µm thick) were obtained using a Leica 1850 cryotome. Brain sections were stored at −80 °C. All animal studies were approved by the Institutional Animal Health Care and Use Committee of the University of California-Irvine.

### 4.4. Aβ Plaque Imaging

To ascertain the presence of Aβ, imaging of post-mortem human brain tissues from AD and CN subjects was carried out using [^3^H]PIB for the frontal cortex, as previously described [31], and [^18^F]Flotaza for the anterior cingulate, as previously described [33]. 

### 4.5. Tau Imaging

For tau imaging of post-mortem human brain tissues from AD and CN subjects, [^125^I]IPPI was used for autoradiographic studies, as previously described [31]. 

### 4.6. NMDA Ionophore Imaging

Brain slices from six subjects, 6 AD and 6 control subjects (frontal cortex and anterior cingulate; AD ages 82–90, SP Stage C and control ages 85–88, SP Stage 0-A) were obtained from Banner Health and sliced (10 mm) using a Leica cryotome. Slides were pre-incubated (10 min in 5 mM Tris/pH 7.4 buffer at 37 °C). They were then incubated in the absence or presence of 10 μM glutamate, glycine or glutamate combinations thereof in Tris/pH 7.4 buffer and [^3^H]MK801 (0.017μCi/cc; American Radiolabeled Chemicals, St. Louis, MO, USA) at 37 °C for 2 h. Non-specific binding was measured using unlabeled MK-801 (10 μM). Post-incubation, slices were washed with 5 mM Tris/pH 7.4 buffer (2 × 5 min), then once with cold buffer (0 °C) for 2 min. The brain sections were air-dried, exposed for 4 weeks to a tritium-sensitive film (Perkin Elmer, Waltham, MA, USA), and then placed on the Phosphor Autoradiographic Imaging System (Packard Instruments Co., Meriden, CT, USA). Using the Optiquant acquisition and analysis program (Packard Instruments Co.), regions of interest (ROIs) were drawn and digital light units/mm^2^ (DLU/mm^2^) were used to quantify the degree of change in [^3^H]MK-801 binding.

### 4.7. Serotonin 5-HT1A Imaging Using [^18^F]Mefway

Purified [^18^F]Mefway was also used for autoradiographic studies of serotonin 5HT-1A receptors in rat brain slices, as previously described [61].

### 4.8. Molecular Docking 

Binding of drugs to the GluN1a/GluN2B NMDA receptor was carried out using our previously described procedures for the Chimera molecular modeling program [31,62]. The molecular structure of the GluN1a/GluN2B NMDA receptor was downloaded from the PDB database [14]. Using Autodock Vina we performed blind docking of energy-minimized molecular structures of MK-801, memantine and other drugs to the NMDA receptor ion channel [31,62]. The grid box dimensions were chosen so that the surface sites in the ion channel were captured. The clusters corresponding to binding energies (represented by Kcal/mol) with the least negative scores were considered for analysis of the different binding sites.

### 4.9. Immunohistochemistry 

Immunostaining of all brain sections was carried out by the pathology services of the University of California-Irvine, using Ventana BenchMark Ultra protocols and analyzed using QuPath, as previously described [34].

### 4.10. Image Analysis 

Group differences between AD and CN subjects were determined using Microsoft Excel 16 and GraphPad Prism 9. Their statistical power was determined using Student’s *t*-test, and *p* values of <0.05 were considered to indicate statistical significance.

## 5. Conclusions

A selective, validated imaging radiotracer suitable for detecting changes in the NMDA receptor ion channel’s opening/availability in the living human brain will be highly significant [15,37,63]. Such an imaging radiotracer can be valuable in clinical programs assessing the use of NMDA antagonists in the treatment of ischemic stroke, brain injury and neurodegeneration. The functionality of the NMDA receptor complex may be altered due to increases in glutamate levels in post-ischemic, post-traumatic and patients with neurodegeneration from the typical ambient extracellular micromolar concentrations of glutamate and glycine [64,65,66]. The increases in synaptic neurotransmitter levels may be 10-fold higher and may be measurable via a suitable imaging radiotracer for the NMDA ion channel for diagnostics and treatment planning. Although many compounds have been shown to possess a nanomolar binding affinity for the NMDA ion channel, the lipophilicity of the compounds has been assumed to be a major issue and detrimental for in vivo imaging. Unlike radiotracers for pre- and postsynaptic receptors and proteins, where the binding of the ligand is considered close to the surface of the cell membrane, the binding site for the ion channel is deeper and glutamate-gated. This requires striking a balance between the hydrophilic and lipophilic character of ion channel radiotracers. With the crystal structure of the NMDA receptor now available (Figure 1 and Figure 2), the binding site of the ion channel is now more clearly understood. A lack of open ion channel “availability” has been one of the major detrimental factors in developing a successful in vivo PET imaging radiotracer for the NMDA receptor ion channel. Our preliminary data support the possibility of an anomalous glutamate and glycine site in the AD brain. More importantly, it appears to show a reduction in the “co-agonist” nature of glutamate and glycine involved in the activation of the NMDA ion channel.

## Data Availability

The data that support the findings of this study are available from the corresponding author upon reasonable request.
